# Antisocial Behavior and Interpersonal Values in High School Students

**DOI:** 10.3389/fpsyg.2017.00170

**Published:** 2017-02-14

**Authors:** María del Mar Molero Jurado, María del Carmen Pérez Fuentes, José J. Carrión Martínez, Antonio Luque de la Rosa, Anabella Garzón Fernández, África Martos Martínez, Maria del Mar Simón Márquez, Ana B. Barragán Martín, José J. Gázquez Linares

**Affiliations:** ^1^Department of Psychology, University of AlmeríaAlmería, Spain; ^2^Department of Psychology, Universidad Autónoma de ChileChile, Chile

**Keywords:** antisocial behavior, interpersonal values, high school students, convivence, profile of subjects

## Abstract

This article analyzes the characteristics of antisocial behavior and interpersonal values of high school students (*Compulsory Secondary Education*) (CSE), the profile of students with high levels of antisocial behavior with regard to interpersonal values, and possible protection from antisocial behavior that interpersonal values could provide. The Interpersonal Values Questionnaire was used to assess interpersonal values, and the Antisocial-Delinquent Behaviors Questionnaire was employed to assess antisocial behaviors. The sample was made up of 885 CSE students aged 14–17. The results revealed a greater prevalence of antisocial behaviors among males and fourth-year CSE students. Moreover, antisocial behaviors were more frequent among participants with high scores in Stimulation, Recognition, Independence, and Leadership and low scores in Conformity and Benevolence. Lastly, logistic regression analyses showed that low scores in Conformity and Benevolence and high scores in Independence predicted high scores in antisocial behavior. The possibility of identifying certain interpersonal values which could positively or negatively affect the appearance of antisocial behavior during adolescence is discussed.

## Introduction

According to Farrington (2005), antisocial behavior is characterized by a style of interpersonal relations seeking group value and recognition, is manipulative and deceitful, lacks empathy, is socially insensitive, impulsive, irresponsible and disobedient. It thus includes “a wide variety of behaviors which reflect violation of societal norms and/or aggression against others" (Kazdin and Buela-Casal, 1996, p. 19). Most authors agree on a series of characteristics that define this type of behavior, such as lack of respect of social norms and the rights of others (Martínez and Gras, 2007), its multifactorial origin (López and Rodríguez-Arias, 2012), and its manifestation linked to the influence of personal variables (such as gender, age, or personality traits) (Pahlavan and Andreu, 2009; Calvete and Orue, 2010; Peña, 2010). As antisocial behavior reaches its maximum expression during adolescence, it becomes of great interest to research on this developmental stage (Pérez-Fuentes et al., 2011; Light et al., 2013; Inglés et al., 2014; Gázquez et al., 2015). Other authors refer to problems in conceptualizing antisocial behavior, differentiating between aggressive forms and rule-breaking (Burt, 2013), since they correlate with different factors, such as proactive aggression (Andreu and Peña, 2013). To the extent that this type of behavior constitutes a risk, not only for the person him/herself, but also for those he/she relates with, effective intervention strategies are necessary not only for its elimination, but also for its prevention (Fernández-Cabezas et al., 2011). Achievement of these goals of intervention and prevention goes through knowledge of antisocial behavior risk and protection factors (López and Rodríguez-Arias, 2012).

## The Origin Of Antisocial Behavior And Socialization Contexts

The early beginning of antisocial behavior (Loeber and Burke, 2011) makes the family, as the child’s first socialization context, of special relevance and importance in the presence of risk and protection factors (Antolín et al., 2009; Álvarez et al., 2015). Some authors suggest childhood abuse or exposure of minors to domestic violence as risk factors for antisocial behavior in adolescence (Sousa et al., 2011), as well as the presence of antisocial behavior of parents, which has a negative impact on the mental and emotional health of their children (Silberg et al., 2012). Murray et al. (2012) found that sudden changes in family structure, such as incarceration of one of the parents, increased the probability of antisocial behavior in their children by 10%.

Furthermore, regarding the family context as a source of possible protective factors against antisocial behavior, Jaureguizar and Ibabe (2012) suggest that the promotion of prosocial attitudes and acquisition of values in the family maintains an inverse relationship with development of antisocial attitudes in children and adolescents. These benefits are also confirmed in the meta-analysis by Piquero et al. (2009), in which the reduction in problematic behavior, including antisocial and delinquent behaviors, was outstanding after application of several types of family intervention going from training in parenting to house visits. Family support not only has positive effects on children who grow up in socially adequate environments, but also performs a protective function in marginal and disfavored environments (Schofield et al., 2012).

Nor should it be forgotten that the educational context provides opportunities for interaction with the peer group, which can be both a system for protection of and risk to young people developing antisocial and delinquent behaviors, and is therefore also relevant to their study (Gázquez et al., 2011). The presence of a positive school environment is therefore a protective factor against both acquisition and maintenance of problematic behavior (Wu et al., 2010). On the other hand, a negative school climate is characterized by the presence of problems with coexistence and bullying, presents higher prevalence of antisocial behaviors and more motivation problems are observed (Rodríguez and Mora-Merchán, 2014; Rodríguez et al., 2014; Regueiro et al., 2015; Valle et al., 2015a,b). Thus the “aggressive victim” responds to harassment by projecting a self-image as rebellious and antisocial (Emler, 2009), while the aggressor is characterized by being highly impulsive (López et al., 2008) and rejecting norms (Povedano et al., 2012). Finally, some authors find a positive correlation between the role of aggressor and antisocial behavior (Cerezo and Méndez, 2013).

## Differences In Manifestation Of Antisocial Behavior: Gender, Age, And School Year

Much of the research on prevalence of antisocial behavior which has analyzed the differences in gender suggests that males show higher rates of antisocial behavior than females (López and Rodríguez-Arias, 2010; Hasking et al., 2011; Viñas et al., 2012). However, although men show more aggressiveness than women (Muñoz et al., 2010), this trend may be changing, since female involvement in violent situations is growing (Pozo, 2012).

Martí and Palma (2010) thought that sex and age have a significant effect on adolescent preferences for values: Girls prefer more abstract, interiorized values and are prone to instrumental values with a more egocentric and material load. As age advances, adolescents prefer values more in harmony with personal dignity and equality rather than those focusing on oneself or on confrontation with others. More recently, Garaigordobil et al. (2014) observed that males involved in bullying tend toward domination and are more aggressive than women.

Regarding the age variable, no one moment has been agreed upon for either appearance of antisocial behavior or its prevalence during an individual’s development. Some have placed its appearance at around 13 years of age (Rechea, 2008), while others like Tresgallo (2011) have suggested that first manifestations appear at 6–7 years, intensifying in late adolescence (Cifuentes and Londoño, 2011), and still others have suggested that it is relatively stable through adulthood (Estévez et al., 2007). More recently, findings in a sample of adolescents aged 13–18 showed that older adolescents exhibit antisocial behavior more often than younger adolescents. Thus the stage of psychological development, and not just age, is of especial importance in the analysis of origin and maintenance of antisocial behavior.

Finally, another of the aspects analyzed is the school year, which is of interest for studying transitions, since this is where the appearance of behavior negative to the school climate becomes most likely (Pellegrini et al., 2010). The prevalence of such behavior in each school year is also studied, because there is a positive correlation between its prevalence and subject age, and also with school year. And the older they are, that is, in higher school years, student justification of violence decreases and is there is more of it among males (Garaigordobil et al., 2013).

## Antisocial Behavior and Interpersonal Values

Interpersonal values are defined as factors determining human behavior, aspects by which each person behaves one way or another when relating to others, depending on their system of values (Gordon, 1979). Thus, throughout development, and especially during adolescence, where interpersonal relations start to become more important, decisions made in different moral dilemmas are of significant importance (Paciello et al., 2013). Therefore, in recent years, several studies associated with this stage of development have analyzed the protective variables avoiding the appearance of antisocial behavior (Inglés et al., 2013) and those which could attenuate its manifestations once they have appeared (Loeber and Farrington, 2012). Thus attitudes and values, such as social sensitivity, prosocial leadership, or security in interpersonal relationships, have been related to social competency in adolescents (Jiménez and López-Zafra, 2011). Other studies have found association of certain interpersonal values and aggressive behavior in the school. Fossati et al. (2012) suggested that low scores in friendliness/benevolence are closely related to participation in violence against schoolmates. According to Georgiou et al. (2013), individualism as a cultural value could be related to an authoritarian style and proneness to intimidation.

After reviewing the literature on the subject in the study in hand, the following research hypotheses were posed: H_1_: More antisocial behavior in males in higher grades; H_2_: Antisocial behavior differs depending on the higher/lower interpersonal value scores; and H_3_: Interpersonal values have different weights on the scale as predictors of antisocial behavior.

In spite of the history of research in antisocial behavior in its role as a predictor of other repertoires of problematic behavior, such as substance abuse (Clark et al., 2002) and its clinical applications (Yakeley and Williams, 2014), this study attempts to clarify the weight of other variables, such as interpersonal values, susceptible to early intervention. Better understanding of these variables during adolescence is essential to progress in research on the causes of these behaviors and for the development of prevention programs addressing antisocial behavior at the youngest ages. Acquisition of interpersonal values is therefore presented as a tool for the prevention of violent behaviors, but unlike the traditional trend of research on the subject, in this case, from a more positive focus. Work is therefore concentrated on identifying those values which make the adolescent a competent social being (Oliva et al., 2010).

This study pursues a better explanation in this regard by analyzing the characteristics of antisocial behavior and interpersonal values based on gender and school year, describing the interpersonal value profile of individuals with high levels of antisocial behavior, and finally, to what extent interpersonal values protect against antisocial behavior.

## Materials and Methods

### Participants

The sample was acquired by random cluster sampling by the different geographic areas [Center, *Levante* (East), and *Poniente* (West)] in the province of Almeria (Spain) from which five public high schools in rural and urban areas were selected at random. Each zone had at least one high school and four classes per school, two in third and two in fourth year of high school, *Educación Secundaria Obligatoria* (*Compulsory Secondary Education*) (ESO), and the sample from each area was over 200 students.

The total sample was made up of 1055 students from 3rd and 4th year of high school of whom 120 were disqualified (11.37%) because they did not finish the questionnaires in time due to their poor mastery of the Spanish language. Another 50 (4.74%) were disqualified due errors or omissions or not having attended one of the two sessions it was given in. Thus the final sample was comprised of a total of 885 students ranging from 14 to 17 years of age, with a mean age of 15.2 years (*SD* = 0.90). Of the total sample, 49.8% (*n* = 441) were males and 50.2% (*n* = 444) were females, with a mean age of 15.22 (*SD* = 0.92) and 15.19 (*SD* = 0.89), respectively. Sample distribution by geographic areas was 212 students (24%) from the center of the province, 333 students from *Levante* (37.6%), and from *Poniente* 340 students (38.4%).

Distribution of the sample by school year was as follows: 3rd year ESO (*n* = 475; 241 males and 234 females) and 4th year ESO (*n* = 410; 200 males and 210 females). The chi-square test for homogeneous distribution of frequencies showed absence of statistically significant differences in gender and school year among the four groups (χ*^2^*_(1,885)_ = 0.34; *p* = 0.56).

### Instruments

*Survey of Interpersonal Values* (SIV; Gordon, 1977). Ninety yes/no items measuring six aspects of the subject’s relations with others:

• Stimulation: Is treated kindly, with consideration and understanding and perceives support from others.
• Conformity: Following the rules, doing what is socially correct, conforming and acting in conformity with what is accepted and suitable.
• Recognition: Being recognized by others, admired and looked up to, attracting positive attention.
• Independence: Doing and considering it one’s right to do whatever one wants, decide for oneself using own criteria and being free.
• Benevolence: Being generous, helping others and doing things for and sharing with them.
• Leadership: Exerting authority over others from a position of command or power.

The Cronbach’s alpha is from 0.78 to 0.89 (Gordon, 1993).

*Antisocial-Delinquent Behaviors Questionnaire* (*A-D*; Seisdedos, 1995). It is comprised of 40 items which assess antisocial (trespassing, littering, etc.) and delinquent behaviors (taking drugs, stealing, etc.). Its reliability and validity are adequate (α = 0.88), as they are in our sample, with a total Cronbach’s alpha slightly above (α = 0.92). In this study, only the antisocial behavior scale was used (α = 0.90).

### Procedure

First, a meeting was held with the directors or counselors at the various schools selected to explain the research goals and inform them of the instruments to be used, as well as to request the permission and cooperation necessary to implement the study. This study was exempt from ethical approval, because the study did not involve any potential risk for the participants. All participants provided written consent. Then the counselors met with and informed the parents of the purpose of the study and requested their consent for the participation of their children. The questionnaires were then administered in two 50-min sessions with an interval between which varied with the school and the class, but was always over 20 min. The questionnaires were coded for their identification by students and to keep them otherwise anonymous. They were administered by groups, voluntarily and anonymously in the classroom or other space at the school if several classes were grouped together. The researchers responsible for the study were present at both the parents meeting and during administration of the questionnaires, to answer questions or resolve doubts, etc.

### Data Analysis

A cross-sectional descriptive design was used for this study in order to analyze the antisocial behavior and interpersonal values by gender and school year and find any relationships between students’ interpersonal values (stimulation, conformity, recognition, independence, benevolence, and leadership) and antisocial behavior.

The Student’s *t*-test was used for the first objective and to find out the mean scores of males and females as well as students in third and fourth year high school on antisocial behavior and interpersonal values (Are there any statistically significant differences in antisocial behavior and interpersonal values between men and women? Are there any statistically significant differences in antisocial behavior and interpersonal behavior between students in 3rd and 4th year?). In addition, to find out the magnitude or effect size of those significant differences indicated by the *t*-test, the Cohen’s *d* was calculated Cohen’s (1988) and interpreted as *d* ≤ 0.20 minimum effect size, *d* = 0.21 a *d* = 0.50 means a small effect size, *d* = 0.51 a *d* = 0.79 means a medium effect size, and when *d* ≥ 0.80 the effect is large.

Identification of the sample on the *SIV Questionnaire* interpersonal value scales (Gordon, 1977) was done when the normal distribution of each had been tested. The thresholds of the scales were differentiated after their normal distribution had been checked. Two groups were formed from the total sample (*N* = 885) for each of the scales: (a) students with low scores in Stimulation, Conformity, Recognition, Independence, Benevolence, and Leadership, that is, those who scored the same or below the 25th percentile (scores equal to or over 14, 11, 8, 13, 14, and 7, respectively) (*N*_2S_ = 233, 26.3%; *N*_2C_ = 235, 26.6%; *N*_2R_ = 218, 24.6%; *N*_2I_ = 237, 26.8%; *N*_2B_ = 262, 29.6%, and *N*_2L_ = 240, 27.1%); (b) students with high scores in Stimulation, Conformity, Recognition, Independence, Benevolence, and Leadership, that is, those who scored the same or over 20, 19, 15, 21, 22, and 14, respectively) (*N*_1S_ = 291, 32.9%; *N*_1C_ = 227, 25.6%; *N*_1R_ = 238, 26.9%; *N*_1I_ = 268, 30.3%; *N*_1B_ = 248, 28%, and *N*_1L_ = 246; 27.8%). This procedure, commonly used in evolutionary psychology, provides two groups with high and low levels. This is also along the same line as other authors who discuss how various facets of antisocial behavior are related to the individual’s social development (Espinosa and Clemente, 2011), so it is important to analyze the scores in antisocial behavior from a group criterion. Subjects with intermediate levels did not form part of the sample analyzed.

The Student’s *t*-test was used to analyze the differences in antisocial behavior between students with high and low scores on the *SIV Questionnaire* scales (Are there any statistically significant differences in antisocial behavior between students with higher/lower scores on each of the interpersonal values scales?). And again, the Cohen’s *d* (Cohen’s, 1988) was calculated to find out the magnitude or effect size of the significant differences shown by the *t*-test.

Aside from this, for the purpose of analyzing the ability of interpersonal values to predict students’ antisocial behavior, a binary logistic regression analysis was performed using forward stepwise regression based on the Wald statistic (What is the predictive value of interpersonal values on antisocial behavior?). Thus two groups were formed, one for the six predictive variables (Stimulation, Conformity, Recognition, Independence, Benevolence, and Leadership) and one for the criterion variable (antisocial behavior), maintaining the one used for the previous test for the predictive variables. For classifying the sample for antisocial behavior, the same criterion was used as in the *SIV Questionnaire* scales, using the following procedure to find out who had high and low scores: (a) students with high Antisocial Behavior, those who scored in the 75th percentile or over (scores the same or over 13) (*N*_1_ = 238, 26.9%); (b) students with low Antisocial Behavior, those who scored in the 25th percentile or below (scores equal to or over 5) (*N*_2_ = 260, 29.4%). This model enables the probability of occurrence of a certain fact or event (e.g., highly aggressive behavior) in the presence of one or several predictors (e.g., high Stimulation, Conformity, Recognition, Independence, Benevolence, or Leadership). This probability is estimated by the odd ratio statistic (OR), both in the total sample and in the samples formed by gender and school year. Statistical analyses were done with the SPSS 20 statistical package.

## Results

### Antisocial Behavior and Interpersonal Values as a Function of Gender and School Year

Observing the mean scores in both Antisocial Behavior and the various interpersonal values by gender, males are observed to have had higher mean scores on the presence of Antisocial Behaviors, Recognition and Leadership, and these were significantly higher than for females, with small effects of gender (*d* ≤ 0.50) on Antisocial Behavior (*d* = 0.24), on Recognition (*d* = 0.44), and Leadership (*d* = 0.38). On the contrary, in Conformity and Benevolence, females showed significantly higher mean scores than males, and again in this case, the effects of gender were small (*d* ≤ 0.50) for Conformity (*d* = 0.15) and Benevolence (*d* = 0.47) values (**Table 1**).

**Table 1:** Difference in means in antisocial behavior and interpersonal values by gender and school year.

	Males			Females			Statistical significance		
	*N*	*M*	*DE*	*N*	*M*	*DE*	*t*	*p*	*d*

AB	441	9.76	5.69	444	8.45	5.26	3.56	0.00	0.24
SIV-S	433	17.25	4.44	437	17.54	4.37	-0.96	0.34	n.s.
SIV-C	426	14.46	5.21	436	15.23	5.38	-2.15	0.03	0.15
SIV-R	431	12.74	4.60	436	10.75	4.42	6.53	0.00	0.44
SIV-I	430	17.27	5.76	434	17.41	5.79	-0.33	0.74	n.s.
SIV-B	429	16.39	5.97	437	19.06	5.48	-6.86	0.00	0.47
SIV-L	430	11.72	5.14	429	9.85	4.64	5.60	0.00	0.38

	**3rd year**			**4th year**			**Statistical significance**		
	***N***	***M***	***DE***	***N***	***M***	***DE***	***t***	***p***	***d***

AB	475	8.50	5.29	410	9.81	5.69	-3.54	0.00	0.24
SIV-S	471	16.97	4.38	399	17.90	4.40	-3.12	0.01	0.21
SIV-C	468	15.93	5.08	394	13.57	5.29	6.65	0.00	0.46
SIV-R	471	11.28	4.42	396	12.29	4.78	-3.22	0.01	0.22
SIV-I	466	17.00	5.88	398	17.74	5.63	-1.87	0.06	n.s.
SIV-B	468	18.34	5.84	398	17.04	5.85	3.26	0.01	0.22
SIV-L	465	10.39	4.77	394	11.25	5.18	-2.50	0.01	0.17

*AB, Antisocial Behavior; SIV-S, support; SIV-C, conformity; SIV-R, recognition; SIV-I, independence; SIV-B, benevolence; SIV-L, leadership; n.s., not significant.*

With regard to school year, the students in the fourth year scored significantly higher than those in third year on Antisocial Behavior, and Stimulation, Recognition and Leadership values, with small effects of the school year variable (*d* ≤ 0.50). On the contrary, students in the third year scored significantly higher in Conformity and Benevolence values than the fourth year, again with small effects of the school year variable (*d* ≤ 0.50).

### Antisocial Behavior in Subjects with High and Low Scores on Interpersonal Values

**Table 2** shows the differences between students with low and high scores on the SIV scales with respect to the presence of Antisocial Behavior for the whole sample and for gender and school year. In the total sample, all the variables are observed to have had significant differences in Antisocial Behavior means. High SIV Stimulation, Recognition, Independence, or Leadership were associated with the presence of higher Antisocial Behavior, while higher Antisocial Behavior means were present in students with low levels of Conformity and Benevolence. The effects of the Conformity (*d* = 0.80), Independence (*d* = 0.74) and Benevolence (*d* = 0.56) scales on Antisocial Behavior were medium (*d* ≥ 0.51), while the Stimulation (*d* = 0.27), Recognition (*d* = 0.21) and Leadership (*d* = 0.28) scales had small effects (*d* ≤ 0.50) on Antisocial Behavior.

**Table 2:** Difference in means in antisocial behavior in students with low and high scores on interpersonal values.

SIV		Low			High			Statistical significance		
Total		*N*	*M*	*SD*	*N*	*M*	*SD*	*t*_522_	*p*	*d*
Antisocial Behavior	*SIV-S*	233	8.25	5.67	291	9.71	5.12	-3.09	0.01	0.27
	*SIV-C*	235	11.64	5.08	227	7.52	5.23	8.57	0.00	0.80
	*SIV-R*	218	8.83	5.48	238	10.03	5.78	-2.27	0.02	0.21
	*SIV-I*	237	7.23	5.07	268	11.08	5.31	-8.32	0.00	0.74
	*SIV-B*	262	10.50	5.68	248	7.50	5.04	6.31	0.00	0.56
	*SIV-L*	240	8.49	5.24	246	10.02	5.72	-3.07	0.01	0.28

**Hombre**		***N***	***M***	***SD***	***N***	***M***	***SD***	***t*_251_**	***p***	***d***

Antisocial Behavior	*SIV-S*	115	9.27	6.01	138	10.13	4.98	-1.21	*n.s.*	–
	*SIV-C*	127	12.21	4.83	105	8.31	5.94	5.40	0.00	0.73
	*SIV-R*	82	9.37	5.80	151	10.31	5.72	-1.19	*n.s.*	–
	*SIV-I*	111	7.87	5.45	133	11.46	5.40	-5.15	0.00	0.66
	*SIV-B*	167	10.56	5.80	91	8.42	5.42	2.89	0.01	0.38
	*SIV-L*	94	9.31	5.67	161	10.40	5.85	-1.45	*n.s.*	–

**Mujer**		***N***	***M***	***SD***	***N***	***M***	***SD***	***t*_269_**	***p***	***d***

Antisocial Behavior	*SIV-S*	118	7.25	5.15	153	9.33	5.23	-3.27	0.01	0.40
	*SIV-C*	108	10.97	5.30	122	6.85	4.43	6.34	0.00	0.85
	*SIV-R*	136	8.51	5.27	87	9.56	5.90	-1.37	*n.s.*	–
	*SIV-I*	126	6.66	4.65	135	10.71	5.20	-6.60	0.00	0.82
	*SIV-B*	95	10.41	5.49	157	6.97	4.74	5.25	0.00	0.68
	*SIV-L*	146	7.95	4.88	85	9.29	5.42	-1.92	*n.s.*	–

**Curso 3∘**		***N***	***M***	***SD***	***N***	***M***	***SD***	***t*_279_**	***p***	***d***

Antisocial Behavior	*SIV-S*	141	7.28	5.37	140	9.54	5.01	-3.65	0.00	0.44
	*SIV-C*	94	11.90	4.71	150	7.26	4.96	7.23	0.00	0.96
	*SIV-R*	131	8.26	5.23	112	9.51	5.63	-1.79	*n.s.*	–
	*SIV-I*	143	6.91	4.87	140	10.86	5.17	-6.61	0.00	0.79
	*SIV-B*	125	9.80	5.78	153	6.73	4.36	4.89	0.00	0.61
	*SIV-L*	143	8.00	4.75	116	8.83	5.70	-1.24	*n.s.*	–

**Curso 4∘**		***N***	***M***	***SD***	***N***	***M***	***SD***	***t*_241_**	***p***	***d***

Antisocial Behavior	*SIV-S*	92	9.73	5.83	151	9.87	5.23	-0.18	*n.s.*	–
	*SIV-C*	141	11.46	5.32	77	8.03	5.71	4.43	0.00	0.63
	*SIV-R*	87	9.69	5.75	126	10.50	5.91	-0.98	*n.s.*	–
	*SIV-I*	94	7.71	5.34	128	11.33	5.46	-4.91	0.00	0.67
	*SIV-B*	137	11.15	5.53	95	8.73	5.79	3.20	0.01	0.43
	*SIV-L*	97	9.20	5.83	130	11.08	5.54	-2.47	0.01	0.33

*SIV-S, support; SIV-C, conformity; SIV-R, recognition; SIV-I, independence; SIV-B, benevolence; SIV-L, leadership; n.s., not significant.*

Gender analysis shows significant differences in mean scores on Antisocial Behavior for the Conformity, Independence and Benevolence scales, and also on the Stimulation scale in females, although its effects were small (*d* = 0.40). In both male and female groups, students with low Conformity and Benevolence showed higher means in Antisocial Behavior, with medium effects of both scales (*d* ≥ 0.51), except in males, where high or low values in Benevolence had small effects (*d* = 0.38) on Antisocial Behavior.

In the analysis of third and fourth years of high school, significant differences are also observed in the mean score on Antisocial Behavior depending on whether the scores on the Conformity, Independence and Benevolence scales were high or low, all of them with a medium effect (*d* ≥ 0.51), except in the fourth year for the last scale, Benevolence (*d* = 0.43), where the effect was small. Furthermore, only in the third year of high school were there differences in the Stimulation scale, and these also had a small effect (*d* = 0.44).

### Are Interpersonal Values Predictors of Antisocial Behavior?

**Table 3** presents the probability of high Antisocial Behavior derived from the binary logistic regression in both the total sample and by gender and school year. It is observed that percentages correctly classified in the total sample vary from 56.5% of the Recognition and Leadership scales to 72.6% of the Conformity scale. The percentages of correct classification by gender go from 62% and 59.2% to 71.9% and 74.1% in males and females, respectively. Finally, in the analysis of the sample by school year, percentages go from 61.1% and 62.9% to 75.4% and 71.3% in the third and fourth year, respectively. In fourth year of high school, the Leadership scale did not form part of the model. The Nagelkerke *R*^2^ varied from 0.02 for the total sample on Recognition and Leadership scales to 0.32 for the third year on the Conformity scale.

**Table 3:** Logistic regression for the probability of high antisocial behavior.

Total	*B*	*SE*	Wald	*p*	OR	IC 95%	*R*^2^ Nagelkerke	% Correct
SIV-S	0.63	0.24	6.86	0.01	1.87	1.17-3.00	0.03	57.7
SIV-C	-1.95	0.28	49.57	0.00	0.14	0.08-0.24	0.26	72.6
SIV-R	0.52	0.24	4.59	0.03	1.68	1.04-2.71	0.02	56.5
SIV-I	1.92	0.27	51.12	0.00	6.81	4.02-11.52	0.25	72.1
SIV-B	-1.34	0.25	29.11	0.00	0.26	0.16-0.43	0.13	65.9
SIV-L	0.53	0.24	4.67	0.03	1.69	1.05-2.72	0.02	56.5

**Male**	***B***	***SE***	**Wald**	***p***	**OR**	**IC 95%**	***R*^2^ Nagelkerke**	**% Correct**

SIV-C	-1.93	0.38	25.79	0.00	0.14	0.07-0.31	0.24	71.9
SIV-I	1.71	0.36	22.12	0.00	5.55	2.72-11.34	0.21	70.3
SIV-B	-0.96	0.35	7.79	0.01	0.38	0.19-0.75	0.07	62.0

**Female**	***B***	***SE***	**Wald**	***p***	**OR**	**IC 95%**	***R*^2^ Nagelkerke**	**% Correct**

SIV-S	0.83	0.35	5.83	0.02	2.30	1.17-4.53	0.05	59.2
SIV-C	-2.07	0.42	23.81	0.00	0.13	0.05-0.29	0.28	73.5
SIV-I	2.20	0.41	29.06	0.00	9.04	4.06-20.14	0.30	74.1
SIV-B	-1.60	0.38	17.37	0.00	0.20	0.09-0.43	0.17	70.4

**3rd year**	***B***	***SE***	**Wald**	***p***	**OR**	**IC 95%**	***R*^2^ Nagelkerke**	**% Correct**

SIV-S	0.87	0.33	6.80	0.01	2.38	1.24-4.56	0.06	61.1
SIV-C	-2.34	0.43	29.56	0.00	0.10	0.04-0.22	0.31	75.4
SIV-I	1.98	0.36	30.16	0.00	7.28	3.58-14.78	0.26	72.8
SIV-B	-1.57	0.36	18.75	0.00	0.21	0.10-0.42	0.17	68.6

**4th year**	***B***	***SE***	**Wald**	***p***	**OR**	**IC 95%**	***R*^2^ Nagelkerke**	**% Correct**

SIV-C	-1.51	0.40	14.22	0.00	0.22	0.10-0.48	0.15	69.8
SIV-I	1.78	0.40	19.38	0.00	5.93	2.68-13.09	0.21	71.3
SIV-B	-0.95	0.36	7.03	0.01	0.39	0.19-0.78	0.07	62.9

*SIV-S, support; SIV-C, conformity; SIV-R, recognition; SIV-I, independence; SIV-B, benevolence; SIV-L, leadership; B, coefficient; SE, standard error; p, probability; OR, odd ratio; IC, interval of confidence at 95%.*

The Odd Ratio’s interpretation of the data from the whole sample shows that the probability of Antisocial Behavior is: (a) 1.87 times higher in students with high Stimulation, (b) 0.14 times lower in students with high Conformity, (c) 1.68 times higher in students with high Recognition, (d) 6.81 times higher in students with high Independence, (e) 0.26 times lower in students with high Benevolence, and (f) 1.69 times higher in students with high Leadership.

In the analysis by gender and school year, the probability of having antisocial behavior is: (a) 0.14 (males), 0.13 (females), 0.10 (third year high school), and 0.22 (fourth year high school) times lower in students with high Conformity, (b) 0.38 (males), 0.20 (females), 0.21 (third year high school), and 0.39 (fourth year high school) times lower in students with high Benevolence, (c) 5.55 (males), 9.04 (females), 7.28 (third year high school), and 5.93 (fourth year high school) times higher in students with high independence, (d) 2.30 (females), and 2.38 (third year high school) times higher in students with high Stimulation.

## Discussion

With regard to the first of the goals of this study, it is observed that males showed higher mean scores on antisocial behavior, coinciding with previous results (López and Rodríguez-Arias, 2010; Hasking et al., 2011; Viñas et al., 2012). Furthermore, in the analysis of interpersonal values, females showed significantly higher mean scores than males in conformity and benevolence with small effects of gender (*d* ≤ 0.50) in both cases.

In the analysis of the influence of school year, its effect was also small, and it was the fourth year students who showed significantly higher scores in Antisocial Behavior, which suggests an increase in frequency of negative, violent or antisocial behavior with year (Cifuentes and Londoño, 2011; Pérez-Fuentes et al., 2011; Garaigordobil et al., 2013). Moreover, in interpersonal values, students in fourth year scored significantly higher in the Stimulation, Recognition and Leadership values than those in third year, while those in third showed higher means in Conformity and Benevolence.

With respect to the study’s second goal, the results show that students who had the highest Antisocial Behavior means, regardless of gender and school year, were those who are treated with kindness, consideration, understanding, etc., who are recognized by others, admired and looked up to, who do what they want and decide for themselves, those who exert authority over those under them, show low conformity (often do not obey the rules or do what is socially correct) and low benevolence (are not generous or do not help others).

Finally, with respect to the third goal, high Stimulation, Recognition, Independence and Leadership values are statistically significant positive predictors of the probability of high scores on Antisocial Behavior, while low scores on Conformity and Benevolence are statistically significant negative predictors of the probability of high scores on Antisocial Behavior.

Logistic regressions by gender and school year were only done for those values in which there were differences in the results of mean interpersonal value scores between the high and low level groups. So low scores on Conformity and Benevolence values and high scores on Independence predict high scores on Antisocial Behavior for both males and females, and both years of high school analyzed. Furthermore, when the Stimulation value was analyzed for females in third year high school, it was also found to be a statistically significant positive predictor of the probability of high scores on Antisocial Behavior.

Limitations of the study are that: (1) It is a sample, which although representative, is comprised only of high school students and cannot be generalized to other grade levels, and therefore, one of the future lines of research is the replication of this study in other years, adapting the questionnaires to be used. (2) The questionnaire used to measure antisocial behavior only gives a total assessment, so it is not possible to find out whether these relationships with interpersonal values are also given for the various different aspects that construct includes, and future research should use a questionnaire such as the Antisocial and Delinquent Behavior Scale (Andreu and Peña, 2013) which allows various factors to be differentiated (predelinquent behavior, vandalism, violence, crimes against property, use of alcohol and drugs). (3) Finally, another of the limitations refers to the biases typical of self-report techniques, such as social desirability. Some cases (Soubelet and Salthouse, 2011) have been found of association of the effects of social desirability, which with age show positive relations with certain desirable characteristics of the self-report and negative with those undesirable.

However, although this study does have some limitations which should be kept in mind for future research, it may be considered a precursor, and is of great interest for the relevant data it contributes to the design of interventions which make it possible to work on reducing risk factors and strengthening those which protect against antisocial behavior at the same time (López and Rodríguez-Arias, 2012). It is recommended that future lines of research include other variables of interest to grouping, such as sociocultural diversity, above all, if that characteristic is representative of the sample. The possibility of adding variables gradually for their analysis makes possible a systematic approach for in-depth study of the subject. The need to continue progressing in the study of the variables involved in antisocial behavior in adolescence therefore emerges from our results.

## Author Contributions

JC, AL, and AB (review scientific language); AM, MS, and JG (writing and literature search); MM (analysis of data); MP and JG (design and review); and AG (changes requested by the reviewers).

## Conflict of Interest Statement

The authors declare that the research was conducted in the absence of any commercial or financial relationships that could be construed as a potential conflict of interest.
